# Classifying knowledge used in complementary medicine consultations: a qualitative systematic review

**DOI:** 10.1186/s12906-022-03688-w

**Published:** 2022-08-06

**Authors:** Kate Davies, Milena Heinsch, Campbell Tickner, Caragh Brosnan, Amie Steel, Gupteswar Patel, Molly Marsh

**Affiliations:** 1 School of Humanities, Creative Industries and Social Sciences, University of Newcastle, Callaghan, Australia; 2 Centre for Brain and Mental Health Priority Research Centre, University of Newcastle, Callaghan, Australia; 3 Faculty of Health, University of Technology Sydney, Sydney, Australia

**Keywords:** Complementary medicine, Patient consultation, Knowledge, Patient-centred, Systematic review

## Abstract

**Background:**

Complementary Medicine (CM) is widely used internationally but there is limited understanding of the forms of knowledge CM practitioners use in their clinical practice and how they use this knowledge in interactions with patients**.** This review aims to synthesise the existing evidence on the forms of knowledge that are mobilised, and the role of this knowledge in the interactions between practitioners and patients during CM consultations. It considered a diverse range of CM practice areas to develop a classification of CM practitioners’ knowledge use in consultations.

**Methods:**

Systematic searches of health and sociology databases were conducted using core concepts, including complementary and alternative medicine, practitioners, and knowledge. Articles were included where they reported on data from recorded CM practitioner and patient consultations and offered insights into the types and applications of knowledge used in these consultations. 16 unique studies were included in the review. Data were extracted, coded and analysed thematically.

**Results:**

Results demonstrate that diverse sources of knowledge were mobilised by practitioners, predominantly derived from the patients themselves –their bodies and their narratives. This reflected principles of patient-centredness. The use of discipline specific forms of knowledge and references to biomedical sources illustrated ongoing efforts towards legitimacy for CM practice.

**Conclusion:**

CM practitioners are navigating tensions between what some might see as competing, others as complementary, forms of knowledge. The classification system provides a useful tool for promoting critically reflective practice by CM practitioners, particularly in relation to self-assessment of knowledge translation and patient interactions.

**Supplementary Information:**

The online version contains supplementary material available at 10.1186/s12906-022-03688-w.

## Background

Complementary Medicine (CM) – defined as a group of diverse medical and health care systems, practices, and products that are not presently considered to be part of conventional medicine [1] – is widely used internationally [2] but there are ongoing debates about the scientific evidence base for CM therapies [3]. CM modalities often work with holistic philosophies that may be viewed as contrasting with biomedical models of knowledge classification, such as those underpinning evidence-based practice [4]. CM practitioners and patients are reported to value patient-centred care, but little is known about how this translates into practitioner-patient communication [5, 6]. Understanding knowledge use in this context is crucial for improving CM practitioner training, patient safety and patient-centred care.

There have been some efforts to understand the relative importance of various types of knowledge in CM practice. In one study CM practitioners reported their most frequently used knowledge sources as: traditional knowledge; textbooks; clinical practice guidelines; published clinical evidence; fellow practitioners; personal intuition; patient preference; personal preference; published experimental evidence; and trial and error [7]. Drawing on interviews with CM providers, Agarwal [8] asserted that CM knowledge is based on historical, cultural and Indigenous systems of belief. There has been no attempt to develop a broader classification of how knowledge is applied in direct communications with patients.

This systematic review examines the extant evidence on the forms and uses of knowledge in interactions between practitioners and patients during a diverse range of CM consultations. Knowledge was defined broadly as comprising information, knowledge and wisdom [9]. As an exploratory systematic review, it draws together qualitative data as a preliminary step towards building depth of understanding on this topic. The review of this qualitative data informs our proposed classification taxonomy, which may aid knowledge translation, evidence-based practice, research uptake and consistent use of terminology [10, 11, 12]. In this review we present a classification of nine sources of knowledge and four domains of knowledge use reported in the literature on CM practice.

## Methods

We followed a method devised by Galbraith et al. [10], comprising three steps: 1) using the literature to identify relevant characteristics (core elements); 2) categorising the core elements based on similarities; and 3) grouping the core elements into broad domains. We used a systematic review of qualitative data to generate a list of knowledge sources (the core elements) which were then categorised according to the application of knowledge (the domains) in CM consultations. A qualitative review was deemed useful in extracting rich, descriptive data as a foundation for building a classification system.

### Searches

The systematic review examined empirical, qualitative data in peer-reviewed literature published between 1 January 2000 and 6 May 2020. The date limits were set in order to examine CM knowledge use within current contexts, particularly in light of the fact that CM use has become more common in many parts of the world. Only articles published in English were included. An updated search was conducted for literature published up to 6 December 2021. Databases searched were AMED, CINAHL, Cochrane Library, Embase, Medline, Proquest and Scopus. Covidence software was used throughout the screening process. The protocol for this systematic review was published on PROSPERO in June 2020 [13]. The search strategy is provided in Appendix A and search strategy results for each database provided in Appendix B.

### Inclusion and exclusion criteria

The qualitative systematic review was designed in accordance with the PICO tool [14, 15]. The population was CM practitioners likely to use individual patient consultations, interest was their use of knowledge in consultations, context was individual consultations with patients and outcomes were forms of, and ways of using, knowledge. Only studies that provided data from recorded consultations were included in order to examine what actually happens rather than reflections on what should or might happen during consultations. Data derived from methods such as practitioner interviews were considered to privilege practitioner interpretations and not offer the real-time examples of knowledge use needed to conceptualise this particular exploratory framework.

### Quality assessment

Article quality was assessed using a nine-point checklist adapted from Hawker et al. [16] and Walsh and Downe [17] that considered the clarity and relevance of information in relation to: abstract and title, introduction and aims, methods and data collection, sampling, data analysis, ethics and bias, results, transferability or generalisability, and implications for practice. Each criterion was scored out of four, with a maximum total score of 36 possible. No papers were excluded based on quality.

### Data extraction

Six reviewers participated in article selection, data extraction, quality assessment and data analysis. KD removed duplicates and papers outside of the date range then conducted the title and abstract screening for all papers. The whole review team discussed criteria for inclusion prior to the title search. KD, GP and CT screened a sample of 20 papers to check the application of the criteria and then KD completed the title and abstract screening. Two reviewers completed full text screening for each article, with KD, GP, CT and CB contributing to this process. Conflicts were discussed by the review team and resolved by a third reviewer where necessary. Data extraction was shared between KD, MH, CT, CB and GP.

### Data synthesis and analysis

A six-step thematic content analysis of the extracted data was conducted by KD and MH [18]. KD and MH individually and inductively generated thematic codes within the categories “sources of knowledge” and “how knowledge was used”. They then cross-checked and consolidated themes and collaboratively categorised thematic findings using the taxonomy developed by Galbraith et al. [10] to produce a classification system. The whole review team provided feedback.

## Results

**Fig. 1:**
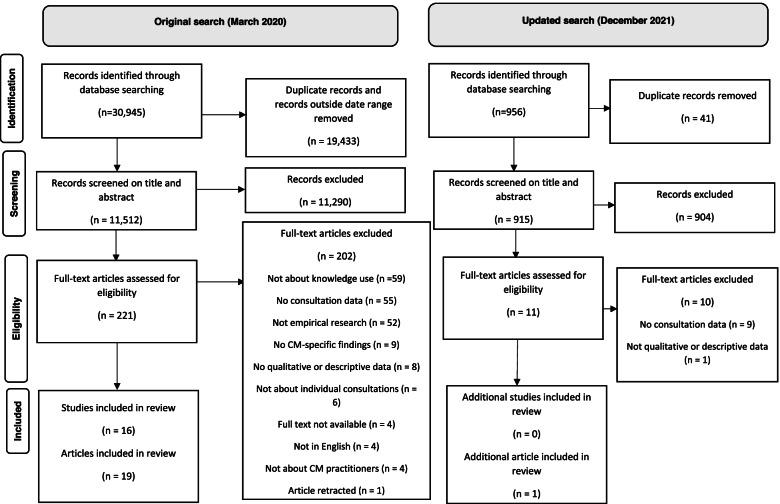
PRISMA flow diagram of study selection

The search returned a higher number of initial results than anticipated. There were numerous papers from health disciplines, such as nursing, that made brief mention of CM, but did not focus on CM practice and there were numerous studies that reported on data other than consultationrecordings. The search results at each stage of review are shown on the PRISMA flow diagram below (Fig. 1).

### Study characteristics

Studies were from eight different countries and nine different CM disciplines were discussed. No studies had considered practitioners’ knowledge across the broad spectrum of CM fields of practice included in this review and as such the findings are unique. For quality assessment, out of a total possible score of 36, four papers scored below 20, 13 scored between 20 and 29 and three papers scored 30 or above. The median score was 25.

There were 20 papers assessed as eligible, reporting on 16 unique studies, as summarised in Table 1 below.

**Table 1:** Study characteristics

Study no.	Author/s (year)	Country	Title of paper	Type of CM practice	Sources of knowledge	Use of knowledge	Study method	Paper quality score
1	Bolton, J. (2000) [19]	USA	Trust and the healing encounter: an examination of an unorthodox healing performance	chiropractic; applied kinesiology	Practice wisdom, patient’s bodies, biomedicine, traditional knowledge	Diagnose and treat, legitimise	Ethnographic case study, observation	12
2	Chant, B. et al. (2017) [20]	Japan	Beliefs and values in Japanese acupuncture: an ethnography of Japanese trained acupuncture practitioners in Japan	acupuncture	Patients’ narratives, formal education and training, traditional knowledge	Diagnose and treat	Qualitative ethnography	23
3	Chatwin, J. (2012) [21]	UK	Damning with faint praise: how homoeopaths talk about conventional medicine with their patients	homeopathy	Biomedicine, patients’ narratives, traditional knowledge	Legitimise	Conversation analysis	20
	Chatwin, J. (2008) [22]		Pre-empting ‘trouble’ in the homoeopathic consultation					18
4	Ciocanel, A. (2016) [23]	Romania	“A remedy that suits me”: Classification of people and individualization in homeopathic prescribing	homeopathy	Patients’ bodies, traditional knowledge, research evidence	Diagnose and treat, legitimise	Qualitative, ethnography, textual analysis	12
5	Eyles, C., Leydon, G. M. & Brien, S. B. (2012) [24]	UK	Forming connections in the homeopathic consultation	homeopathy	Practice wisdom, patients’ narratives, biomedicine, intuition	Relate, diagnose and treat, legitimise	Qualitative, data collected via interviews, observations and practitioner diaries, grounded theory	29
	Eyles, C. et al. (2011) [25]		A grounded theory study of homeopathic practitioners’ perceptions and experiences of the homeopathic consultation					26
6	Fortune, L. D. & Hymel, G. M. (2015) [26]	USA	Creating integrative work: a qualitative study of how massage therapists work with existing clients	massage therapy	Practice wisdom, intuition, biomedicine, formal education and training, patients’ narratives	Relate, diagnose and treat	Qualitative, ethnomethodology, hermeneutic phenomenology	28
7	Hennius, B. J. (2013) [27]	UK	Contemporary chiropractic practice in the UK: a field study of a chiropractor and his patients in a suburban chiropractic clinic	chiropractic	Patients’ narratives, traditional knowledge, patients’ bodies, intuition, biomedicine	Diagnose and treat, legitimise, educate and inform	Ethnography	29
8	Ho, E.Y. & Bylund, C.L. (2008) [28]	USA	Models of health and models of interaction in the practitioner-client relationship in acupuncture	acupuncture/TCM	Patients’ narratives, traditional knowledge, biomedicine	Diagnose and treat, legitimise, relate	Ethnography	20
9	Paterson, C. et al. (2012) [29]	UK	Communication about self-care in traditional acupuncture consultations: the co-construction of individualised support and advice	acupuncture	Patients’ bodies, traditional knowledge	Diagnose and treat, educate and inform	Qualitative, constructivist, conversational analysis	21
10	Pun, J., Chor, W. & Zhong, L. (2019) [30]	Hong Kong	Delivery of patient-centered care in complementary medicine: Insights and evidence from the Chinese medical practitioners and patients in primary care consultations in Hong Kong	TCM	Patients’ bodies, patients’ narratives, traditional knowledge	Diagnose and treat, education and inform	Conversation/discourse analysis	28
	Pun, J.		Moments of ‘touch’ as a way for mental support in Traditional Chinese Medicine consultations: Analysis of the interactional process of co-constructing understanding of the patient’s body conditions in Hong Kong					24
11	Ruusuvuori, J. (2005) [31]	Finland	Comparing homeopathic and general practice consultations: the case of problem presentation	homeopathy	Patients’ narratives, traditional knowledge, biomedicine	Legitimise, diagnose and treat	Comparative analysis of General Practitioner and Homeopath consultations	21
12	Segar, J. (2012) [32]	UK	Complementary and alternative medicine: exploring the gap between evidence and usage	homeopathy; acupuncture; reiki	Personal experience, biomedicine	Legitimise	Qualitative	28
13	Stöckigt, B.M. et al. (2015) [33]	Germany	Healing relationships: a qualitative study of healers and their clients in Germany	spiritual healing; meditation	Intuition, personal experience, patients’ narratives	Relate, diagnose and treat, legitimise	Qualitative study using observation and interviews with spiritual healers and clients	30
14	Stub, T., Foss, N. & Liodden, I. (2017) [34]	Norway	“Placebo effect is probably what we refer to as patient healing power”: a qualitative pilot study examining how Norwegian complementary therapists reflect on their practice	Chiropractic, acupuncture, naturopathy, homeopathy	Personal experience, practice wisdom, patients’ bodies	Relate, diagnose and treat, legitimise	Social qualitative fieldwork, anthropology	14
15	Thomson, O. P.; Petty, N. J. & Moore, A. P. (2014) [35]	UK	A qualitative grounded theory study of the conceptions of clinical practice in osteopathy - a continuum from technical rationality to professional artistry	osteopathy	Traditional knowledge, practice wisdom, patients’ bodies, patients’ narratives, formal education and training	Diagnose and treat, legitimise, educate and inform	Qualitative, constructivist grounded theory	25
	Thomson, O. P., Petty, N.J. & Moore, A.P. (2014) [36]		Clinical decision-making and therapeutic approaches in osteopathy - a qualitative grounded theory study					34
16	West, V. & Denham, A. (2017) [37]	UK	The clinical reasoning of Western herbal practitioners: A qualitative feasibility study	western herbal practice	Traditional knowledge, patients’ narratives, intuition, practice wisdom	Diagnose and treat	Qualitative case study	30

### Sources of knowledge

All included articles reported that CM practitioners used multiple sources of knowledge during consultations with patients. We describe the main sources here and note others that were mentioned briefly.

#### The patient’s narrative

The most common source of knowledge came from patients themselves. The studies indicated that CM practitioners were often guided by the preferences and values expressed by their patients. Stories shared by patients were noted as sources of knowledge in seven papers [21, 24, 25, 27, 30, 31, 37]. These were collected via patients’ self-reports (verbal or written) and based on accumulated understanding of the patients’ histories over time [21, 25, 27]. Some CM practitioners prioritised time for patients to share their stories in order to gather information and facilitate a collaborative approach to knowledge. Eyles et al. [24] reported that “practitioners considered that the process of narrative exploration often seemed to assist the patient in engaging with homeopathic principles”. Connecting and building rapport with patients was a means of generating knowledge and part of a holistic conceptualisation of what counts as knowledge [25]. The knowledge derived from listening to, and understanding, a patient was described by some practitioners as fundamental to a mutual relationship in which the patient’s expertise about their own health and body sat in tandem with practice wisdom [28].

From observations of Traditional Chinese Medicine (TCM) consultations, Pun et al. [30] concluded that eliciting patient’s histories was a distinguishing feature of patient-centred care, noting that practitioners “were observed to skilfully create space for patients to express their concerns and to offer opportunities for patients to seek advice through social talks”. Knowledge gleaned from a patient’s history demonstrated an individualised approach to consultation [26]. Ruusuvuori [31] found that the patients’ narratives informed diagnosis and treatment and connections to homeopathic knowledge frames, noting that:

In her narrative, the patient foregrounds the link between her life-history, her present lifeworld situation and her present symptoms … thus indicating that she orients to the homeopathic ideas on holism and the importance of her own individual perspective [31].

However, the extent to which practitioners prioritised getting to know patients and their preferences varied. Thomson et al. [35, 36] found that osteopaths who subscribed to biomedical modes of knowledge were not particularly interested in patients’ stories. In contrast, those osteopaths who prioritised patients’ stories were deemed to exhibit professional artistry and one such osteopath reported that “I like spending time talking to the person about what’s going on and how it’s impacting them” [36].

#### The patient’s body

Some practitioners built their knowledge of patients through interacting with patients’ bodies. Observing and touching the body were tools for diagnosis or testing [19]. Working through various modalities of the body, such as respiratory or digestive systems built an understanding the individual [23, 27]. Thomson et al. [35] found that physical indicators were important for some osteopaths, with one such practitioner describing that “I use my palpation to assess and let the body tell me what it wants me to do, and will permit me to do”. A hands-on approach to clinical assessment reflected an intuitive type of tactile knowledge [27]. Pun et al. [30, 38] found that TCM practitioners elicited extensive information from patients about their bodily functions such as sleep, bowel movements and drinking water – knowledge of the patient’s body was embedded in the traditions of TCM consultation. Paterson et al. [29] recorded acupuncturists building this knowledge of the individual patient’s body in silence.

#### Intuition and empathy

Stöckigt et al. [33] found that practitioners who identified as “spiritual healers” drew out knowledge of clients’ stories in non-verbal ways, which the authors described as intuition and empathic understanding. The practitioner’s knowledge of the client came from “fusion” whereby healers reported feeling what clients felt and sharing sensations. One healer stated that, “If somebody says nothing, you hear what he wants to say” [33]. Empathic connections between clients and practitioners were observed to be related to spiritualism as a particular body of knowledge. One practitioner stated that “You can call it also collective unconsciousness or the higher self” [33].

Herbal practitioners reported reliance on intuition within their patient consultations [37]. One practitioner described intuition as “the application of insight” and another as “more about intuition of what he was feeling” [37]. The authors further noted difficulties distinguishing intuition from tacit knowledge – tacit knowledge being correlated to clinical experience or practice wisdom (discussed below) [37].

#### Traditional knowledge

For many practitioners, traditional forms of CM-discipline knowledge framed the information shared with patients. Paterson et al. [29] described practitioners tailoring advice to patients on the basis of Chinese medicine such as the theory of Yin and Yang. Practitioners in one study reported that TCM consultations followed five traditional steps – wang (inspection), wen (auscultation & olfaction), wen (inquiring), qie (palpation) and zhen (diagnosis) [30].

In contrast, one study found that the chiropractic practitioner privileged contemporary chiropractic theories, particularly where the wisdom of traditional theories had been questioned over time [27]. The author noted that:

Although a unifying idea of chiropractic has been sought since the end of the nineteenth century it has been suggested that there may in fact not exist one concept that would encompass and accommodate the entire chiropractic profession [27].

Knowledge – in terms of theories, principles and texts – was described in discipline-specific terms. In some cases this was reflected in *how* the practitioner communicated. Chatwin [22] identified that homeopathic theory was the underpinning body of knowledge used to explain treatments. Practitioners talked about diagnosis and recommendations specifically in relation to homeopathy principles [21, 22].

Thomson et al. [35, 36] referred to the “dogma” of osteopathy and considered osteopaths’ positions on the practice continuum in relation to how they engaged with theories of osteopathy. They considered that study participants who adhered stringently to standard principles of osteopathy reflected technical rationality, illustrated by one participant who stated that “You need to keep pure to osteopathic philosophy [and] the principles of osteopathy make me do what I do” [36]. Those who were more critical and interpretive in their application of osteopathic theories were deemed to exhibit professional artistry and be more patient-centred during consultations.

Concepts of mysticism and healing reflected a body of knowledge that was distinct from experience or evidence-informed knowledge:

He [chiropractor and applied kinesiologist] may also introduce statements that are not immediately supported by his experience nor by other scientific statements and have a mystical or quasi-natural valence [19].

The term holism appeared in most articles and was often conceptualised as the frame in which knowledge was produced. Holism was described as a strength of CM practice because it accounts for multiple knowledge forms. One acupuncturist stated:

The patient–practitioner are in one circle and that is contained within another larger circle of all the factors that influence a person’s well-being (diet/emotions/exercise) and that’s within the circle of the universe—the energy of the seasons/place/people etc. [28]

In one study the CM practitioner’s knowledge was informed by the specific teachings a particular “guru” [19].

#### Biomedicine

Conventional medical knowledge was, in a way, a yardstick against which evidence was measured, whether positing CM knowledge in opposition to, or as supported by, medical evidence [21]. Bolton [19] found that chiropractors and kinesiologists drew on medical science modes of knowledge to validate diagnoses and treatment recommendations.

Biomedical forms of knowledge were also critiqued. In one study, acupuncturists were critical of the limitations of biomedical evidence for preventing illness [28]. The authors reflected that:

The core assumptions and beliefs of the biomedical view of health and Chinese medicine are extremely different, most notably because the biomedical model does not recognize the essential Chinese medical theories of *Qi*, energetic organs, and yin and yang … [28]

Pun et al. [30] noted that TCM practitioners “moved away from biomedical talk to a broader discussion of the patient’s daily habits and symptoms” and that, in comparison to western medicine practitioners they relied less on “medical apparatus and instruments”. They concluded that doctor-patient interactions were more equal in TCM consultations compared to western medicine consultations. Ruusuvouori [31] compared homeopathy consultations to general practice consultations. Findings from this study disproved the author’s hypothesis that practitioners and patients would be more problem-oriented in medical consultations than homeopathy consultations. However, some homeopaths did resist the problem-orientation by shifting conversation towards a holistic knowledge frame [31].

Segar [32] found that CM therapists and patients were unexpectedly pragmatic, tending to view CM and evidence-based medicine as complementary. Thomson et al. [35, 36] found that half of the 12 osteopaths in their study privileged biomedical knowledge, describing diagnosis and treatment in terms of physiology rather than emotion or psychology. One osteopath said:

If you don’t have the basics like anatomy and physiology you are never going to get the right decision [36].

#### Practice wisdom

CM practitioners were seen to develop knowledge over the course of their professional career [24, 25]. This was observed to evolve into a type of intuition, whereby the practitioner’s practical experience was privileged over other sources of knowledge such as formal training [27].

Stub et al. [34] found that therapists emphasised the importance of their professional skills and therapeutic competence in building relationships with their patients. They identified that complementary therapists understood the placebo effect as the patient’s self-healing power, resulting from establishing trust and belief in the treatment process and the practitioner’s competence.

Thomson, et al. [35, 36] reported that osteopaths who practiced professional artistry were critically reflective about their practice knowledge. These practitioners derived confidence from their own practice wisdom to critique other forms of knowledge. One such osteopath said, “I try to draw on my experience and knowledge and to try and give a balanced opinion” [35].

In exploring the reasoning processes of herbal practitioners, West and Denham [37] found that previous professional experience was an important source of knowledge. One practitioner stated, “If I’m on familiar territory, like hay fever, I’m not going to start looking things up” [37]. The authors referred to tacit knowledge – what they described as “subconscious cognitive knowledge”, aligning with practice wisdom, whereby practitioners can make judgements that feel intuitive because those judgements have been practiced over time [37].

#### The personal experiences of the practitioner

CM practitioners’ personal, non-professional, experiences and observations were sources identified in three studies. One homeopath said that “It’s that internal process of working on yourself and making clear what’s yours and what’s someone else’s” [24]. Another homeopath in this study reported that her personal experience convinced her of efficacy of certain treatments, “I don’t understand how it can work, but I’ve *seen* it work” [24]. Similarly, Stöckigt et al. [33] found that “healers’ own experiences of crisis, illness, and self-healing” shaped their views of practice and the ways they connected with and related to clients.

#### Formal education or training

In one study it was noted that there was a homogenous body of knowledge applied by practitioners (in this case Japanese acupuncturists), because all practitioners were trained within very standardised educational institutions [20].

Formal training was a way to gain knowledge but was not always as well-regarded as other sources [26]. Thomson et al. [35] suggested that the level of a practitioner’s formal education correlated with the approach to knowledge in practice. They observed that osteopaths with graduate degree-level qualifications tended to adhere fairly stringently to what they had learned in their formal education. One such participant noted that “I still use the principles that I was taught as a student...” [35]. In contrast, they found that osteopaths with additional postgraduate qualifications adopted a more critical perspective in which they interpreted practice wisdom, patient preference and discipline-specific principles to inform practice. Homeopaths in Ciocănel’s [23] study reported that textbooks from formal education were useful sources for discipline-specific knowledge.

#### Other sources of knowledge

Sources of knowledge less frequently reported, that are nevertheless enlightening for understanding the spectrum of knowledge, included informal discussions with other CM practitioners, such as discussions outside of the formal parts of seminars and workshops [23] and broad reading, including media, related to public CM debates [32]. Only one study explicitly referred to published research evidence, where a homeopath described a research study to a patient during a discussion about public funding for CM therapies [22].

### Modes of knowledge use

Different CM practitioners drew on different sources of knowledge to achieve the same purpose. There were four recurring themes in the application of knowledge in consultation settings.

#### Relate and centre the patient

For most practitioners the use of knowledge was a relational process guided by the client’s narrative – their “life history, present lifeworld situation, and present symptoms” [31]. Many practitioners emphasised the importance of adopting an individual approach to each session. This entailed talking with clients before and after treatment to determine their expectations and engage them as active participants in the healing process [25–, 26, 27, 28].

Relationships with clients were built up over time, sometimes years, resulting in a high level of trust [33]. In some studies, practitioners described the use of self in combination with a client-centred, narrative approach to develop a profound and unique connection with clients, which facilitated clients’ openness to CM practice [24, 30, 37, 38].

In contrast, practitioners in one study tended not to encourage client involvement, with one practitioner noting that “I don’t want to spend time using words and wasting valuable time, when I can get on with the job and try to achieve my goal” [35]. In these situations, clinical decisions were often based on knowledge gained through interaction with patients’ bodies rather than patients’ values and preferences [35].

#### Justify and legitimise practice and decisions

Several studies found that CM practitioners employed knowledge to justify and legitimise their practice decisions. For example, practitioners tended to refer to professional qualifications, research evidence and contemporary theory to establish trustworthiness and credibility of their disciplines [19, 22, 27]. In one case, medical knowledge was even misapplied to discredit biomedicine [19].

Practitioners used various techniques to frame their practice. In many cases, practitioners used the notion of holism to smooth the way for the introduction of more uncommon concepts such as “energy” and “fluids” [28, 31]. Practitioners reported that physical connections with patients were key to reassuring patients about their competence with one therapist reporting that “I touch them, I control the treatment in a way so that I show that I know what I’m doing” [34]. Some practitioners found that getting to know the patient and their story was a useful means to “assist the patient in engaging with homeopathic principles” [24].

#### Diagnose and treat

Patients’ narratives and bodies were vital to diagnosis and treatment. Bolton [19] described how a chiropractic and kinesiology practitioner’s lengthy process of patient observation and testing often led to a recommendation for further appointments and administration of a supplement. Thomson et al. [36] observed that diagnosis, treatment and management of patients was the singular focus for some osteopaths when applying discipline-specific knowledge.

Getting to know patients was key for some practitioners in forming a diagnosis. One chiropractor noted the need to “ask time and again till you get a clear picture of the symptoms and the underlying causes” [34]. The patient’s physical body could be a primary source of information for formulating diagnosis and treatment. This included observational tests for diagnosis [19, 29] and hands-on assessment to identify the most effective treatments [27].

#### Educate and inform

Some practitioners treated knowledge as a resource to be shared with patients for the purpose of educating and informing them. Thomson et al. [35] described an iterative process whereby practitioners learned from their patients and also imparted knowledge so that “patients were active and informed decisions-makers about their treatment and management” [35]. Paterson et al. [29] similarly observed that TCM practitioners engaged patients in an “interactive discussion” that informed advice about self-care strategies on matters such as diet and exercise.

Hennius [27] noted that, over a number of consultations, one chiropractor used physical assessment, patient narratives and practice wisdom to build the patients’ own knowledge of how to “get better”. The chiropractor applied knowledge to treat the patient *and* enable the patient to care for themselves. Similarly, Pun et al. [30] considered that for TCM practitioners the relational and informative applications of knowledge were interrelated. In talking to and getting to know patients, practitioners were able to understand patients’ concerns while providing an environment in which patients could confidently seek advice.

### Core elements and domains

From thematic analysis of the qualitative data, eight common elements were identified, each representing a source of knowledge that was applied during clinical consultations: patients’ narratives, intuition, personal experience, traditional knowledge, biomedicine, practice wisdom, patients’ bodies and formal education and training. A notable omission is research evidence, which was only briefly mentioned in one study. It is, nevertheless, included as a ninth element given the contentious nature of evidence within this field and as an area that requires further research. Core elements and domains identified in each article and study are set out in Appendix B.

CM practitioners used a variety of knowledge sources to achieve the goals of building relationships with patients, legitimising their practice, making diagnosis and treatment decisions and informing and educating patients. The knowledge sources (core elements) have been mapped against the various purposes for which they were applied in each of the included studies (domains), as shown in Table 2 below.

**Table 2:** CM knowledge classification system

Domains (Purpose of the knowledge in the consultation setting)	Core elements (Source of the knowledge used in the consultation setting)
Relate	Patients’ narratives Intuition Traditional knowledge Personal experience
Legitimise	Personal experience Traditional knowledge Biomedicine Practice wisdom Research evidence
Diagnose and treat	Patients’ narratives Intuition Traditional knowledge Practice wisdom Patients’ bodies Formal education and training Biomedicine
Educate and inform	Patients’ narratives Patients’ bodies Practice wisdom Traditional knowledge

## Discussion and conclusions

### Discussion

The results show that diverse sources of knowledge were mobilised by practitioners to achieve patient-centredness and legitimacy for CM practice. Patient-centredness has been identified as a central tenet of many CM systems [5] and has been reported by patients to increase empowerment and disclosure [6, 39]. However, there is little evidence on the elements of the consultation dynamics which have led to this experience [40]. The studies included in this review suggest practitioners that place a stronger focus on biomedicine may conduct consultations in a manner that diminishes patient-centredness, a concern that has been raised from within CM [41]. From the findings, patient narratives were used to understand patient preferences, in accordance with the evidence-based medicine paradigm [41] and also as sources of knowledge to inform practitioner decision-making; a finding supported through recent international research [42]. In light of this, a closer examination on the interplay between evidence-based medicine, patient-centred care and CM clinical care approaches is warranted.

This review identified that the application of traditional knowledge was, at times, complicated by indistinct boundaries and tensions between experiential, scientific and traditional knowledge, challenges not unique to CM [43]. The concept of the “art” vs the “science” of clinical practice is debated in other health professions [44, 45, 46] but may be amplified due to the importance placed on traditional knowledge within CM systems [2, 3]. CM practitioners are challenged with adapting practice in response to emergent scientific evidence while remaining paradigmatically aligned with the philosophies and principles underpinning the CM system they practice [2, 47].

Our review found that CM practitioners used knowledge in ways common to all health profession clinical practices – such as diagnosis and treatment – as well as to educate their patients and justify decisions. Patient education is a key feature of some CM professions, such as naturopathy [48]. However, this needs to be considered in the context of practitioners potentially using knowledge to legitimise decisions. There is a risk of erroneous use of knowledge through these education activities. This is further amplified in circumstances where CM practitioners are using intuition as knowledge, a finding supported through previous research [49]. Intuition is a complex term which may describe high-order cognitive skills and experiences [50] but also has substantive limitations [51]. CM practitioners also perceive tensions between different knowledge sources [52] and as such further research is needed to explore their approach to integrating diverse knowledge sources to inform clinical decision-making and patient communication. Our team is taking this next step in a qualitative study of CM practitioners’ decision-making and communication, currently underway in Australia.

### Limitations

This review does not capture quantitative measures of knowledge as it is intended as an exploratory starting point on which quantitative and/or mixed-methods reviews might be founded. The exclusion of papers not published in English is a limiting factor and we acknowledge that there are important papers published in languages other than English given the international relevance of this topic.

There is not a definitive list of practices that constitutes CM. What might be considered complementary in some countries is part of mainstream health services in others. This study took a broad view of CM practice, basing its list of included practices on the Cochrane CM therapies search filter, modified to only include practices that predominantly involved individual client consultations [53].

The study focused specifically on CM and did not attempt to draw comparisons with conventional medicine. An equivalent systematic review on knowledge use in conventional medicine would be a helpful next step and would enable a broad comparison between the two domains.

### Conclusions

This review has found CM practitioners use diverse sources of knowledge within clinical encounters and in doing so may operationalise patient-centredness. It also highlights tensions and complexities around the modes of knowledge use in CM practice, particularly where the application of different forms of knowledge and evidence is contested. The review is limited by the exclusion of non-English papers and constrained by the inconsistencies in the definitions of CM used globally. Despite this, our proposed CM knowledge classification system may provide a useful framework for future research investigating CM knowledge use within CM clinical encounters. It may also be a useful tool for critically reflective practice, particularly in relation to self-assessment of knowledge translation and patient interactions. Overall, our knowledge framework can contribute to informed practice and policy debate by shaping consistent approaches to accountability and the evaluation of CM knowledge.

## Supplementary Information

**Additional file 1: Appendix A.** Search strategy. **Appendix B.** Search strategy results. **Appendix C.** Core elements and domains identified in each study.

## Data Availability

The datasets used and/or analysed during the current study are available from the corresponding author on reasonable request.
