# Supplementary material for: Classifying knowledge used in complementary medicine consultations: a qualitative systematic review
Source: BMC Complement Med Ther. 2022 Aug 6;22:212. doi: 10.1186/s12906-022-03688-w (PMC9356449; doi:10.1186/s12906-022-03688-w)
Supplement: Supplementary file 1 — Additional file 1: Appendix A. Search strategy. Appendix B. Search strategy results. Appendix C. Core elements and domains identified in each study. [file 12906_2022_3688_MOESM1_ESM.docx]

**Appendix A. Search strategy**

| **Key Terms** | **Alternate Terms** |
| --- | --- |
| Complementary and alternative medicine | CM OR “complementary medicine” OR “alternative medicine” OR “complementary and alternative medicine” OR naturopath* OR “Chinese medicine” OR chiropract* OR acupuncture OR herbal* OR “complementary therapy” OR reflexology OR aromatherapy OR osteopathy OR “energy therapy” OR “bowen therapy” OR “polarity therapy” OR reiki OR “healing touch” OR “therapeutic touch” OR homeopath* OR massage OR kinesiolog* OR ayurved* |
| Practitioners | Practitioner* OR therapist* OR healer* OR expert* OR professional* OR clinician* OR naturopath* OR chiropractor* OR acupuncturist* OR herbalist* OR reflexologist* OR aromatherapist* OR osteopath* OR homeopath* OR kinesiologist* |
| Knowledge | Evidence OR inform* OR communicat* OR knowledge |

**Inclusion/exclusion criteria:**

| **Inclusion** | **Exclusion** |
| --- | --- |
| Publication date 2000 (inclusive) to present | Publication pre-2000 |
| English language | Non-English language |
| Empirical studies (qualitative, quantitative and mixed methods) | Dissertation/theses |
| Position/discussion/theoretical papers | Conference proceedings |
| Peer-reviewed articles | Published abstracts |
| Literature reviews (narrative, scoping and systematic) | Grey literature/not published in peer reviewed journal |
| Used consultations as a source of data | Did not include any component of consultation data |

**Appendix B. Search strategy results**

Note: Results shown for first search on 6^th^ May 2020. Search was repeated to identify new literature on 6^th^ December 2021.

| **Database** | **Records retrieved** |
| --- | --- |
| AMED | 2452 |
| CINAHL | 7544 |
| Cochrane Library | 204 |
| Embase | 6100 |
| Medline | 7290 |
| Nursing and Allied Health (Proquest) | 2805 |
| Scopus | 4550 |
| **TOTAL** | **30945** |

Database(s): **Ovid MEDLINE(R) and Epub Ahead of Print, In-Process & Other Non-Indexed Citations and Daily**1946 to May 04, 2020

| **#** | **Searches** | **Results** |
| --- | --- | --- |
| 1 | complementary therapies/ or acupuncture therapy/ or homeopathy/ or medicine, chinese traditional/ or therapeutic touch/ or naturopathy/ or reflexotherapy/ or aromatherapy/ | 55204 |
| 2 | Herbal Medicine/ | 2115 |
| 3 | Massage/ | 6054 |
| 4 | Kinesiology, Applied/ | 308 |
| 5 | Medicine, Ayurvedic/ | 2212 |
| 6 | Manipulation, Chiropractic/ | 987 |
| 7 | (CAM or "complementary medicin*" or "alternative medicin*" or naturopath* or "chinese medicin*" or chiropract* or acupuncture or herbal* or "complementary therap*" or reflexology or aromatherap* or osteopathy or "energy therap*" or "bowen therap*" or "polarity therap*" or reiki or "healing touch" or "therapeutic touch" or homeopath* or massage or kinesiolog* or ayured*).tw,kw. | 137835 |
| 8 | 1 or 2 or 3 or 4 or 5 or 6 or 7 | 161774 |
| 9 | (Practitioner* or therapist* or healer* or expert* or professional* or clinician* or naturopath* or chiropractor* or acupuncturist* or herbalist* or reflexologist* or aromatherapist* or osteopath* or homeopath* or kinesiologist*).mp. | 967413 |
| 10 | (Evidence or inform* or communicat* or knowledge).mp. | 3947403 |
| 11 | 8 and 9 and 10 | 8996 |
| 12 | limit 11 to (english language and yr="2000 -Current") | 7525 |
| 13 | abstracts/ or comment/ or editorial/ or letter/ or news/ or published erratum/ or academic dissertation/ or "book review"/ or meeting abstract/ | 2096575 |
| **14** | **12 not 13** | **7290** |

Database(s): **Embase**1947 to present

| **#** | **Searches** | **Results** |
| --- | --- | --- |
| 1 | *alternative medicine/ | 22000 |
| 2 | *acupuncture/ | 24537 |
| 3 | *homeopathy/ | 4842 |
| 4 | *Chinese medicine/ | 18831 |
| 5 | *aromatherapy/ | 833 |
| 6 | *herbal medicine/ | 9454 |
| 7 | *massage/ | 4460 |
| 8 | *kinesiology/ | 729 |
| 9 | *chiropractic manipulation/ | 68 |
| 10 | (CAM or "complementary medicin*" or "alternative medicin*" or naturopath* or "chinese medicin*" or chiropract* or acupuncture or herbal* or "complementary therap*" or reflexology or aromatherap* or osteopathy or "energy therap*" or "bowen therap*" or "polarity therap*" or reiki or "healing touch" or "therapeutic touch" or homeopath* or massage or kinesiolog* or ayured*).ti. | 79048 |
| 11 | 1 or 2 or 3 or 4 or 5 or 6 or 7 or 8 or 9 or 10 | 112659 |
| 12 | (Practitioner* or therapist* or healer* or expert* or professional* or clinician* or naturopath* or chiropractor* or acupuncturist* or herbalist* or reflexologist* or aromatherapist* or osteopath* or homeopath* or kinesiologist*).tw. | 1130839 |
| 13 | (Evidence or inform* or communicat* or knowledge).tw. | 4872739 |
| 14 | 11 and 12 and 13 | 6218 |
| 15 | abstract report/ or editorial/ or erratum/ or letter/ or note/ | 2730779 |
| **16** | **14 not 15** | **6100** |

Database(s): **CINAHL**

| **#** | **Query** | **Results** |
| --- | --- | --- |
| S1 | (MH "Alternative Therapies") OR (MH "Aromatherapy") OR (MH "Homeopathy") OR (MH "Naturopathy") OR (MH "Acupuncture") OR (MH "Manipulation, Chiropractic") OR (MH "Bowen Technique") OR (MH "Reiki") OR (MH "Polarity Therapy") OR (MH "Medicine, Ayurvedic") OR (MH "Medicine, Herbal") | 81,872 |
| S2 | (MH "Massage") | 15,574 |
| S3 | (MH "Applied Kinesiology") | 439 |
| S4 | TI ( (CAM or "complementary medicin*" or "alternative medicin*" or naturopath* or "chinese medicin*" or chiropract* or acupuncture or herbal* or "complementary therap*" or reflexology or aromatherap* or osteopathy or "energy therap*" or "bowen therap*" or "polarity therap*" or reiki or "healing touch" or "therapeutic touch" or homeopath* or massage or kinesiolog* or ayured*) ) OR AB ( (CAM or "complementary medicin*" or "alternative medicin*" or naturopath* or "chinese medicin*" or chiropract* or acupuncture or herbal* or "complementary therap*" or reflexology or aromatherap* or osteopathy or "energy therap*" or "bowen therap*" or "polarity therap*" or reiki or "healing touch" or "therapeutic touch" or homeopath* or massage or kinesiolog* or ayured*) ) | 84,997 |
| S5 | S1 OR S2 OR S3 OR S4 | 137,071 |
| S6 | TI ( (Practitioner* or therapist* or healer* or expert* or professional* or clinician* or naturopath* or chiropractor* or acupuncturist* or herbalist* or reflexologist* or aromatherapist* or osteopath* or homeopath* or kinesiologist*) ) OR AB ( (Practitioner* or therapist* or healer* or expert* or professional* or clinician* or naturopath* or chiropractor* or acupuncturist* or herbalist* or reflexologist* or aromatherapist* or osteopath* or homeopath* or kinesiologist*) ) | 519,448 |
| S7 | TI ( (Evidence or inform* or communicat* or knowledge) ) OR AB ( (Evidence or inform* or communicat* or knowledge) ) | 1,118,033 |
| S8 | S5 AND S6 AND S7 | 7,557 |
| S9 | (MH "Theses and Dissertations") OR (MH "Book Reviews") OR (MH "News") | 23,752 |
| **S10** | **S8 not S9** | **7,544** |

Database(s): **COCHRANE LIBRARY**

**ID Search Hits**

#1 MeSH descriptor: [Complementary Therapies] this term only 336

#2 MeSH descriptor: [Acupuncture Therapy] this term only 2901

#3 MeSH descriptor: [Homeopathy] this term only 232

#4 MeSH descriptor: [Medicine, Chinese Traditional] this term only 912

#5 MeSH descriptor: [Therapeutic Touch] this term only 129

#6 MeSH descriptor: [Naturopathy] this term only 20

#7 MeSH descriptor: [Reflexotherapy] this term only 31

#8 MeSH descriptor: [Aromatherapy] this term only 227

#9 MeSH descriptor: [Herbal Medicine] this term only 59

#10 MeSH descriptor: [Massage] this term only 1074

#11 MeSH descriptor: [Kinesiology, Applied] this term only 52

#12 MeSH descriptor: [Medicine, Ayurvedic] this term only 88

#13 MeSH descriptor: [Manipulation, Chiropractic] this term only 126

#14 (CAM or "complementary medicin*" or "alternative medicin*" or naturopath* or "chinese medicin*" or chiropract* or acupuncture or herbal* or "complementary therap*" or reflexology or aromatherap* or osteopathy or "energy therap*" or "bowen therap*" or "polarity therap*" or reiki or "healing touch" or "therapeutic touch" or homeopath* or massage or kinesiolog* or ayured*):ti,ab 26470

#15 {OR #1-#14} 28050

#16 (Practitioner* or therapist* or healer* or expert* or professional* or clinician* or naturopath* or chiropractor* or acupuncturist* or herbalist* or reflexologist* or aromatherapist* or osteopath* or homeopath* or kinesiologist*).ti,ab 3984

#17 (Evidence or inform* or communicat* or knowledge).ti,ab 4150

**#18 {AND #15, #16, #17}**

Database(s): **Nursing and Allied Health (Proquest)**

noft((CAM or "complementary medicin*" or "complementary therap*" or "alternative medicin*" or naturopath* or "chinese medicin*" or chiropract* or acupuncture or herbal* or "complementary therap*" or reflexology or aromatherap* or osteopathy or "energy therap*" or "bowen therap*" or "polarity therap*" or reiki or "healing touch" or "therapeutic touch" or homeopath* or massage or kinesiolog* or ayured*)) AND noft((Practitioner* or therapist* or healer* or expert* or professional* or clinician* or naturopath* or chiropractor* or acupuncturist* or herbalist* or reflexologist* or aromatherapist* or osteopath* or homeopath* or kinesiologist*)) AND noft((Evidence or inform* or communicat* or knowledge))

Database(s): **SCOPUS**

TITLE ( ( cam OR "complementary medicin*" OR "complementary therap*" OR "alternative medicin*" OR naturopath* OR "chinese medicin*" OR chiropract* OR acupuncture OR herbal* OR "complementary therap*" OR reflexology OR aromatherap* OR osteopathy OR "energy therap*" OR "bowen therap*" OR "polarity therap*" OR reiki OR "healing touch" OR "therapeutic touch" OR homeopath* OR massage OR kinesiolog* OR ayured* ) ) AND TITLE-ABS ( ( practitioner* OR therapist* OR healer* OR expert* OR professional* OR clinician* OR naturopath* OR chiropractor* OR acupuncturist* OR herbalist* OR reflexologist* OR aromatherapist* OR osteopath* OR homeopath* OR kinesiologist* ) ) AND TITLE-ABS ( ( evidence OR inform* OR communicat* OR knowledge ) ) AND ( LIMIT-TO ( DOCTYPE , "ar" ) OR LIMIT-TO ( DOCTYPE , "re" ) )

Database(s): **AMED (Allied and Complementary Medicine)**1985 to April 2020

| **#** | **Searches** | **Results** |
| --- | --- | --- |
| 1 | complementary therapies/ or acupuncture therapy/ or herbalism/ or homeopathy/ or naturopathy/ or therapeutic touch/ or traditional medicine chinese/ | 28594 |
| 2 | Reflexology/ | 241 |
| 3 | Aroma therapy/ | 658 |
| 4 | Massage/ | 2548 |
| 5 | Applied kinesiology/ | 226 |
| 6 | Ayurvedic medicine/ | 816 |
| 7 | Chiropractic/ | 6704 |
| 8 | (CAM or "complementary medicin*" or "alternative medicin*" or naturopath* or "chinese medicin*" or chiropract* or acupuncture or herbal* or "complementary therap*" or reflexology or aromatherap* or osteopathy or "energy therap*" or "bowen therap*" or "polarity therap*" or reiki or "healing touch" or "therapeutic touch" or homeopath* or massage or kinesiolog* or ayured*).tw. | 62253 |
| 9 | 1 or 2 or 3 or 4 or 5 or 6 or 7 or 8 | 64464 |
| 10 | (Practitioner* or therapist* or healer* or expert* or professional* or clinician* or naturopath* or chiropractor* or acupuncturist* or herbalist* or reflexologist* or aromatherapist* or osteopath* or homeopath* or kinesiologist*).mp. | 58296 |
| 11 | (Evidence or inform* or communicat* or knowledge).mp. | 47457 |
| 12 | 9 and 10 and 11 | 2822 |
| 13 | limit 12 to journal article | 2452 |

**Appendix C. Core elements and domains identified in each study**

| **Study** | **Core elements**  (Sources of knowledge) | **Domains**  (Purpose and use of knowledge) |
| --- | --- | --- |
| 1 | Practice wisdom, patient’s bodies, biomedicine, traditional knowledge | Diagnose and treat, legitimise |
| 2 | Patients’ narratives, formal education and training, traditional knowledge | Diagnose and treat |
| 3 | Biomedicine, patients’ narratives, traditional knowledge | Legitimise |
| 4 | Patients’ bodies, traditional knowledge, research evidence | Diagnose and treat, legitimise |
| 5 | Practice wisdom, patients’ narratives, biomedicine, intuition | Relate, diagnose and treat, legitimise |
| 6 | Practice wisdom, intuition, biomedicine, formal education and training, patients’ narratives | Relate, diagnose and treat |
| 7 | Patients’ narratives, traditional knowledge, patients’ bodies, intuition, biomedicine | Diagnose and treat, legitimise, educate and inform |
| 8 | Patients’ narratives, traditional knowledge, biomedicine | Diagnose and treat, legitimise, relate |
| 9 | Patients’ bodies, traditional knowledge | Diagnose and treat, educate and inform |
| 10 | Patients’ bodies, patients’ narratives, traditional knowledge | Diagnose and treat, education and inform |
| 11 | Patients’ narratives, traditional knowledge, biomedicine | Legitimise, diagnose and treat |
| 12 | Personal experience, biomedicine | Legitimise |
| 13 | Intuition, personal experience, patients’ narratives | Relate, diagnose and treat, legitimise |
| 14 | Personal experience, practice wisdom, patients’ bodies | Relate, diagnose and treat, legitimise |
| 15 | Traditional knowledge, practice wisdom, patients’ bodies, patients’ narratives, formal education and training | Diagnose and treat, legitimise, educate and inform |
| 16 | Traditional knowledge, patients’ narratives, intuition, practice wisdom | Diagnose and treat |
